# The mutual interplay between calcification and coccolithovirus infection

**DOI:** 10.1111/1462-2920.14362

**Published:** 2018-09-18

**Authors:** Christopher T. Johns, Austin R. Grubb, Jozef I. Nissimov, Frank Natale, Viki Knapp, Alwin Mui, Helen F. Fredricks, Benjamin A. S. Van Mooy, Kay D. Bidle

**Affiliations:** ^1^ Department of Marine and Coastal Sciences Rutgers University New Brunswick NJ 08901 USA; ^2^ University of South Carolina Honors College Columbia SC 29208 USA; ^3^ Department of Marine Chemistry and Geochemistry Woods Hole Oceanographic Institution Woods Hole MA 02543 USA

## Abstract

Two prominent characteristics of marine coccolithophores are their secretion of coccoliths and their susceptibility to infection by coccolithoviruses (EhVs), both of which display variation among cells in culture and in natural populations. We examined the impact of calcification on infection by challenging a variety of *Emiliania huxleyi* strains at different calcification states with EhVs of different virulence. Reduced cellular calcification was associated with increased infection and EhV production, even though calcified cells and associated coccoliths had significantly higher adsorption coefficients than non‐calcified (naked) cells. Sialic acid glycosphingolipids, molecules thought to mediate EhV infection, were generally more abundant in calcified cells and enriched in purified, sorted coccoliths, suggesting a biochemical link between calcification and adsorption rates. In turn, viable EhVs impacted cellular calcification absent of lysis by inducing dramatic shifts in optical side scatter signals and a massive release of detached coccoliths in a subpopulation of cells, which could be triggered by resuspension of healthy, calcified host cells in an EhV‐free, ‘induced media’. Our findings show that calcification is a key component of the *E. huxleyi*‐EhV arms race and an aspect that is critical both to the modelling of these host–virus interactions in the ocean and interpreting their impact on the global carbon cycle.

## Introduction

Globally distributed, unicellular coccolithophores have existed in the oceans for at least 209–220 million years (Falkowski *et al*., 2004; Monteiro *et al*., 2016), playing prominent roles in the oceanic carbon cycle due to their ability to both photosynthetically fix CO_2_ into particulate organic carbon (POC) and biomineralize particulate inorganic carbon (PIC) as calcium carbonate (CaCO_3_)(Iglesias‐Rodriguez *et al*., 2002). Coccolithophores presently account for at least half of the 80–120 Tmol of annual PIC production in the pelagic ocean (Degens and Ittekkot, 1986; Westbroek *et al*., 1993; Balch *et al*., 2007; Berelson *et al*., 2007; Broecker and Clark, 2009). Coccolith‐associated calcite that is produced in the surface waters is exported into the deep ocean, accounting for ~ 83% of global, ballasted POC fluxes to the deep sea in part because it is more dense and experiences less water column dissolution than opal, and it is more abundant than terrigenous material (Klaas and Archer, 2002). It accounts for up ~ 50% of calcite raining down on marine sediments, with the other 50% derived from foraminifera (Broecker and Clark, 2009).

Export of CaCO_3_ is facilitated by its high density (2.7 g cm^−3^), inherent protective organic coatings (Hassenkam *et al*., 2011), association with particulate organic matter [marine snow, transparent exopolymeric particles and faecal pellets (Pedrotti *et al*., 2012; Collins *et al*., 2015)] and supersaturation in the upper water column (Westbroek *et al*., 1993; Balch *et al*., 2007; Berelson *et al*., 2007; Broecker and Clark, 2009). While there are no coccolith‐specific estimates, the total PIC sinking‐flux below 2000 m may be as much as 50 Tmol C year^−1^ (Berelson *et al*., 2007), with associated CaCO_3_:POC ratios (or ‘rain ratios’) having important implications for POC transport to the deep ocean and biological pump efficiency (Armstrong *et al*., 2002; Klaas and Archer, 2002; Ridgwell *et al*., 2009). Consequently, the interplay between ecosystem processes and calcification is of prominent importance to the fate of POC and PIC, by influencing the relative balance between carbon export (via vertical sinking flux) and attenuation (through lysis and microbial respiration) (Bidle, 2015; Collins *et al*., 2015; Laber *et al.*, 2018).

Given its aforementioned importance to the marine carbon cycle, cellular processes and environmental factors that influence calcite formation and the degree of coccolithophore calcification have received intensive focus (Raven and Crawfurd, 2012), with impacts of ocean acidification garnering more recent attention (Doney *et al*., 2009; Hurd *et al*., 2009; Ridgwell *et al*., 2009; Kroeker *et al*., 2010; Moolna and Rickaby, 2012). Decreases in both calcification and CaCO_3_:POC ratios are generally observed when cells are exposed to CO_2_ levels higher than ambient atmospheric concentrations (390 ppm), while the opposite appears to be true at low CO_2_ concentrations (~ 190 ppm), such as occurred at the last glacial maximum [18,000 years ago; (Riebesell *et al*., 2000; Zondervan *et al*., 2001; Casareto *et al*., 2009)].

While the cellular roles of calcification remain speculative, one hypothesized function is to protect coccolithophores from grazers and virus infection (Nejstgaard *et al*., 1994; Monteiro *et al*., 2016; Raven and Waite, 2004). Experimental evidence with heterotrophic dinoflagellates is mixed with both support for (Hansen *et al*., 1996) and against differential grazing rates on calcified *Emiliania huxleyi* cells, with demonstrated strain‐specific differences in ingestion, independent of calcification. These microzooplankton predators had slower growth rates and gross growth efficiencies when feeding on calcified strains relative to non‐calcified (naked) strains, which when applied to a growth rate model, resulted in the net accumulation of *E. huxleyi* (Harvey *et al*., 2015). To date, the impact of calcification on virus infection is largely unexplored.

Globally distributed blooms of *E. huxleyi*, which span ~ 100,000 km^2^ (Brown and Yoder, 1994; Brown, 1995; Tyrell and Merico, 2004), are often terminated by infection of lytic, double‐stranded DNA containing coccolithoviruses (EhVs) (Bratbak *et al*., 1993; Schroeder *et al*., 2003; Vardi *et al*., 2012). Infection triggers cell lysis and the release of dissolved organic carbon (DOC) and PIC‐laden coccoliths in surface waters along with the production of transparent expolymeric particles (TEP; Passow *et al*., 2001; Passow, 2002; Lehahn *et al*., 2014), which facilitate particle aggregation, high zooplankton grazing and greater downward vertical fluxes of both POC and PIC from the upper mixed layer (Laber *et al*., 2018; Sheyn *et al*., 2018; Nissimov *et al*., 2018). *Emiliania huxleyi*–EhV interactions are mechanistically regulated by a lipid‐based, chemical arms race, with three structurally distinct membrane glycosphingolipids (GSLs) – host GSLs (hGSLs), virus GSLs (vGSLs) and sialic acid GSLs (sGSLs) – at the core of this interaction, each serving a unique diagnostic indicator of different aspects of the infection process (Fulton *et al*., 2014; Bidle, 2015). Present evidence suggests that hGSLs are unique to *E. huxleyi* cells and specifically trace host dynamics (Vardi *et al*., 2012); vGSLs regulate infection by inducing PCD of host cells and are incorporated into EhV virions (Vardi *et al*., 2009). Hence they serve as markers of active infection and EhV production. sGSLs appear to be determinants and promising biomarkers of infectivity and have a proposed, yet unestablished, relationship to PIC (Fulton *et al*., 2014; Hunter et al. 2015).

Surprisingly, little attention has been paid to the interplay of calcification and EhV infection. Successful infection requires adsorption of viruses onto host cells at the cell membrane (Mackinder *et al*., 2009). While this process is not yet resolved in the *E. huxleyi–*EhV system, there is evidence that this initial interaction may take place between specific protein receptors in host lipid rafts [e.g. Toll interleukin 1 receptor (TIR) and leucine rich repeat (LRR) domain proteins] and EhV membrane proteins (i.e., C‐type lectin containing proteins) (Rose *et al*., 2014). Cellular properties and processes that interfere with that interaction, such as enhanced TEP production and/or calcification, may serve to reduce adsorption and regulate infection. A rigorous comparison of adsorption rates and infection dynamics of EhVs for naked and calcified *E. huxleyi* cells has not been conducted.

We tested the relative susceptibility to infection of several calcifying *E. huxleyi* strains, some of which were isolated in 2008 from a mesocosm experiment off the coast of Norway (Vardi *et al*., 2012), at different calcification states. By acclimating strains to different Ca^2+^ concentrations (Herfort *et al*., 2004; Trimborn *et al*., 2007), we specifically tested whether reduced cellular PIC content within the same host strain could impact EhV infection and associated GSL dynamics. This experimental set‐up and approach allowed us to explore *E. huxleyi*–EhV ecological dynamics across alternate calcification states. Our results highlight a component of the *E. huxleyi*–EhV arms race at the interface of the biomineral (coccolith) and EhVs, that can act as a first order control on successful adsorption and infection. EhVs may ostensibly navigate this host protection through the induced manipulation of host calcification state, while the host coccosphere (and associated coccoliths) acts as a potential mechanism to sequester EhVs, both serving to impact ecosystem dynamics and carbon biogeochemistry.

## Results and discussion

### Calcification state and infectivity of *E. huxleyi* strains

**Figure 1:**
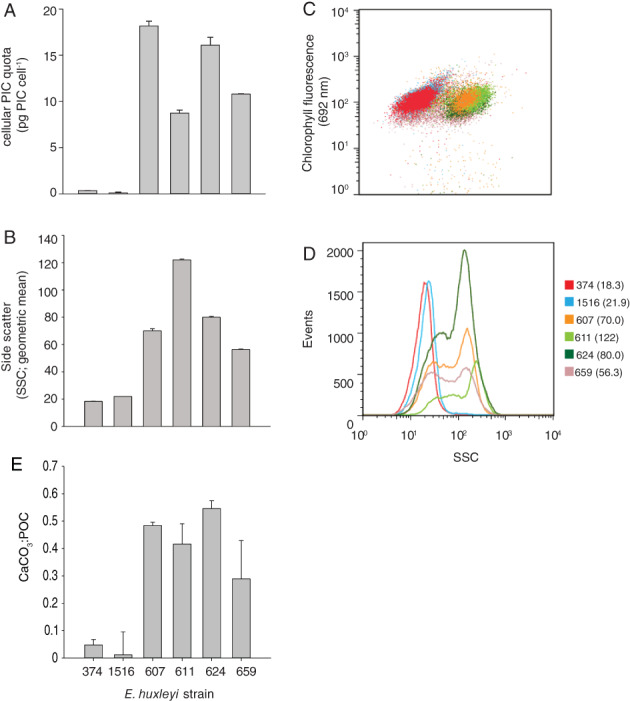
Calcification state of *E. huxleyi* strains grown in replete f/2‐Si media. (A) Cellular PIC quota, (B–D) side scatter (SSC) and (E) CaCO_3_:POC ratios were used as different proxies of calcification. SSC is represented in a variety of ways in order to show the geometric mean of the population (B), its relationship to cellular chlorophyll (692 nm; C) across strains and its frequency distribution across a range of SSC values. Strains are colour coded in panels (C) and (D). Numbers in parentheses correspond to the geometric mean in SSC. CaCO_3_:POC ratios in (E) are derived from data shown in (A). Error bars in (A), (B) and (E) represent the standard error for triplicate measurements (SD/$\sqrt{n)}$.

The *E. huxleyi* strains used in this study (see ‘Experimental procedures’ section) span a range of calcification states, side‐scatter and CaCO_3_:POC ratios relevant to carbon flux (Fig. 1). At 18.2 and 16.1 pg CaCO_3_ cell^−1^, DHB607 and DHB624 possessed the highest cellular PIC quotas in replete f/2‐Si growth media of all strains tested (Fig. 1A). Strains DHB611 and DHB659 were also calcified but displayed more moderate cellular PIC quotas, while both CCMP374 and CCMP1516 had extremely low quotas (Fig 1A and Supporting Information Fig. S10), consistent with previous reports that they are naked strains (Bidle *et al*., 2007; Bidle and Kwityn, 2012). Naked and calcified cells differed in their equivalent spherical diameters (ESDs), measuring ∼3.8 and 4.3 μm respectively. Cellular calcification states were also verified visually using scanning electron microscopy (SEM; Supporting Information Fig. S2) and optically using flow cytometry‐based side scatter (SSC; Fig. 1B–D). All strains had similar chlorophyll fluorescence (Fig. 1C), but the SSC distributions of the calcified strains were clearly distinct from the naked strains. Cultures of calcified cells had a bimodal SSC distribution (Fig. 1D), indicative of a population substructure among low and high SSC cells while the naked strains, CCMP374 and CCMP1516, had single SSC populations. The correlation between SSC geometric means and PIC cell quota across all strains was not significant (Spearman rank order; *P* > 0.05), due in part to the broad SSC distribution in calcified cells. In addition, detached coccoliths would be captured and included in PIC measurements, thereby over‐estimating the cellular PIC quota. In contrast, they are effectively excluded from cellular SSC measurements, given SSC are taken for a population of cells falling within a gated population of chlorophyll containing cells. Despite the lack of clear strain to strain correlation between cellular PIC quotas and SSC signatures, the latter allowed for a rapid, *in situ* interrogation of the changes in cellular calcification in response to EhVs and Ca^2+^ concentrations for a single strain (described below). Corresponding CaCO_3_:POC ratios for these strains ranged from 0.04 to 0.55 (Fig. 1E), consistent with previous ratios reported for *E. huxleyi* (Blanco‐Ameijeiras *et al*., 2016).

**Figure 2:**
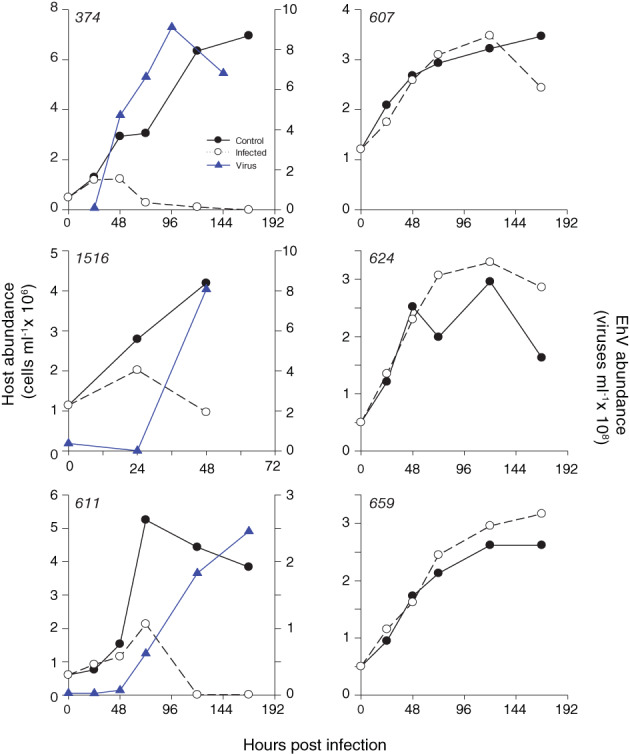
Host–virus infection dynamics for *E. huxleyi* strains grown in replete f/2‐Si media. Time course of host (circles) and virus (blue triangles) abundance for uninfected (closed circles; solid line) and EhV86‐infected (open circles; dotted line). No EhV86 production was detected for strains DHB607, DHB624 and DHB659, indicative of resistance. Host–virus dynamics shown were representative of experiments (*n* = 3) performed on different dates.

*Emiliania huxleyi* host strains displayed distinct differences in host–virus infection dynamics when challenged with the coccolithovirus type strain EhV86 (Fig. 2). Both naked strains, CCMP374 and CCMP1516, were highly sensitive to EhV86 infection, consistent with previous observations (Schroeder *et al*., 2002; Bidle *et al*., 2007; Vardi *et al*., 2009; Bidle and Kwityn, 2012; Fulton *et al*., 2014; Kendrick *et al*., 2014) with cell abundance declining within 48–72 h post infection (hpi) concomitant with high levels of virus production (~ 9 × 10^8^ viruses ml^−1^). Consequently, these naked strains served as positive controls for sensitivity to infection. Conversely, with the exception of DHB611, all calcified strains displayed resistance to EhV86 infection, with infected cultures largely tracing the dynamics of uninfected control cells and lacking measurable EhV production. While DHB611 displayed sensitivity to EhV86, as evidenced by virus production and host cell lysis after ~ 72 hpi, infection was less intense; only 7.32 × 10^6^ viruses ml^−1^ were produced at 48 hpi compared to considerably higher EhV production (4.73 × 10^8^ viruses ml^−1^) and more rapid cell lysis observed for the highly sensitive, naked strain CCMP374 (Fig. 2).

### Manipulating calcification state and host–virus dynamics

**Figure 3:**
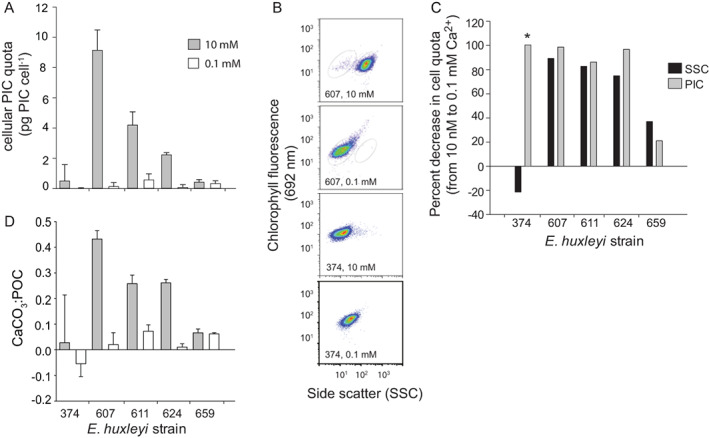
Calcification state of various *E. huxleyi* strains grown in defined ESAW media at different Ca^2+^ concentrations. (A) Cellular PIC quota for strains grown with either 0.1 mM or 10 mM Ca^**2+**^ concentrations. Error bars represent the standard error (SD/$\sqrt{n)}$ among triplicate measurements for one experiment; host–virus dynamics shown were representative of experiments (*n* = 3) performed on different dates. (B) Representative flow cytometry plot showing the SSC and chlorophyll (692 nm) signals for DHB607 grown in 10 mM or 0.1 mM Ca^**2+**^ compared to canonical naked strain CCMP374 grown in 10 mM Ca^**2+**^. Note the prominent shift to a lower SSC for DHB607 cells grown in 0.1 mM, matching that of the low SSC for CCMP374. (C) Percentage (%) decrease in both the mean cellular PIC quota and geometric mean of SSC for cells grown in 10 mM Ca^2+^ compared to those grown in 0.1 mM Ca^2+^ (asterisk for strain CCMP374 denotes that the % decrease in PIC derives from a 0 pg PIC cell^−1^ at 0.1 mM Ca^2+^). (D) CaCO_3_:POC ratios derived from the data in panel (A). We noted that growth in the ESAW artificial seawater medium at 10 mM Ca^2+^ did generally lower the total calcification state for all DHB strains compared to cells grown in f/2‐Si media (Figs 1A and 3A), likely due to the presence of additional factors in seawater‐based media that are required for maximum calcification. Nonetheless, these strains clearly calcified in ESAW media, maintained similar growth rates, and possessed similar cellular chlorophyll signatures, indicative of healthy cells. SEM images confirmed that there was plenty of calcite production in culture under these conditions, with the formation of fully formed coccospheres and detached coccoliths (Supporting Information Fig. S2).

We acclimated the aforementioned calcifying strains to low calcium (0.1 mM Ca^2+^) and compared them to cells grown in 10 mM Ca^2+^ (i.e., ambient concentration in seawater; Tyrrell and Zeebe, 2004) in a defined growth media (ESAW; see ‘Experimental procedures’ section). Measurements of cellular PIC quotas, SSC signatures and CaCO_3_:POC ratios of acclimated cultures confirmed dramatic reductions in calcification state for strains grown at 0.1 mM Ca^2+^ (Fig. 3). This was especially evident for DHB607, where PIC cell^−1^, SSC signatures and CaCO_3_:POC ratios dropped by 90%, 98% and 95%, respectively. Flow cytometry confirmed that DHB607 cells grown at different Ca^2+^ concentrations had distinct SSC signatures with those grown in 0.1 mM Ca^2+^ having similar SSC signatures to that of naked CCMP374 cells (Fig. 3B). The other calcified strains, especially DHB611 and DHB624, also showed significant reductions in both PIC quotas and SSC; DHB659 had similarly low cellular PIC quotas at both Ca^2+^ concentrations. While no significant change was detected in the PIC cell^−1^ for naked strain CCMP374 (Fig. 3A and C), it did show a ~ 20% increase in SSC (Fig. 3B and C), which has been previously documented and attributed to changes in the cell membrane during acclimation to low calcium concentrations (Von Dassow *et al*., 2012).

This method of acclimating cells to different calcium concentrations was previously shown to effectively lower cellular PIC, while allowing a similar growth physiology (identical growth rates and culture dynamics; Herfort *et al*., 2004; Trimborn *et al*., 2007). It also allowed us to avoid the acute treatment of cells with Ca^2+^ chelators such as EDTA, which have also been used to remove coccoliths (de Jong *et al*., 1976; Lyon, 2014). Such treatment could artificially impact host physiology and host–virus interactions over the 96 h infection period. Individual strains maintained similar levels of cellular fluorescence, as assessed by flow cytometry. Across biological replicates, strains DHB607, DHB624 and DHB659 (*P* < 0.05, *n* = 3) had statistically indistinguishable specific growth rates under both Ca^2+^ concentrations (Supporting Information Fig. S1). DHB611 was replicated twice and showed similar specific growth rates in both conditions. While statistically significant differences were observed for CCMP374 (*P* < 0.05, *n* = 3), specific growth rates were very similar (*μ* of 0.70 *vs* 0.65 day^−1^; Supporting Information Fig. S1).

**Figure 4:**
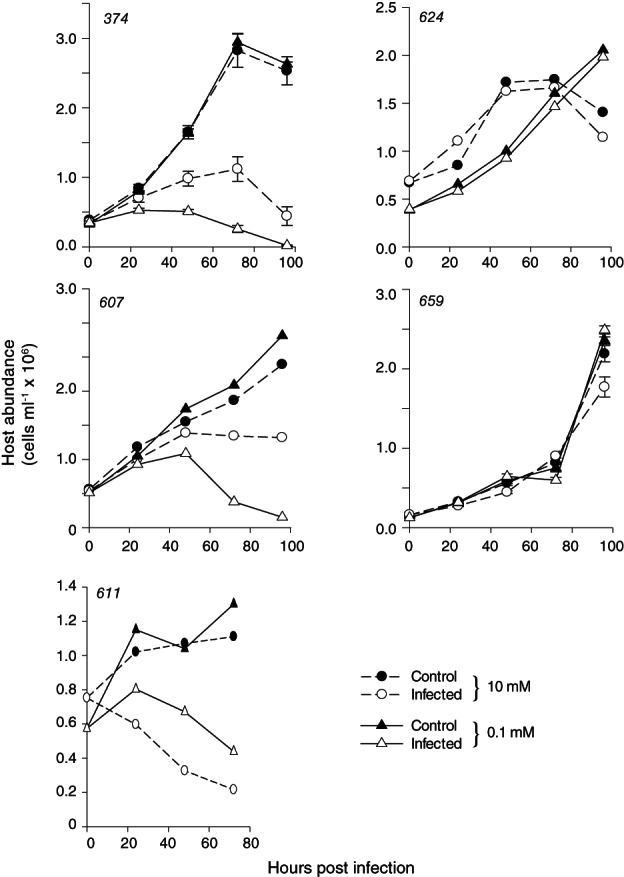
Host–virus infection dynamics for various *E. huxleyi* strains at different calcification states. Time course of host abundance for uninfected (closed circles; solid line) and EhV86‐infected (open circles; dotted line) *E. huxleyi* cells that were grown in ESAW with either 0.1 or 10 mM Ca^**2+**^. Data for EhV86 production for strains that showed evidence of infection (CCMP374, DHB607 and DHB611) are shown in Supporting Information Fig. S3; no EhV86 production was detected for strains DHB624 and DHB659. Error bars represent the standard deviation among triplicate measurements for one experiment; host–virus dynamics shown without error bars were representative of experiments (*n* = 3 for DHB607 and DHB624; *n* = 2 for DHB611) performed on different dates. Where not visible, error bars are smaller than symbol size.

Calcium concentrations also had a pronounced effect on infection dynamics in three calcified strains: CCMP374, DHB607 and DHB611 (Fig. 4 and Supporting Information Fig. S3). Earlier and more pronounced reductions in host cell abundance (Fig. 4), concomitant with more rapid EhV production (Supporting Information Fig. S3) for CCMP374 and DHB607 cells acclimated to lower Ca^2+^ levels, demonstrated an elevated sensitivity to EhV infection in these strains. Notably, control incubations at both Ca^2+^ concentrations paralleled each other for all strains so these differences were not due to differential growth rates under these conditions.

Both DHB607 and DHB611 cells grown in 0.1 mM Ca^2+^ were characterized by prominent host cell lysis and steady declines in cell abundance after 48 and 24 hpi respectively. These strains both showed evidence of infectivity at both 0.1 and 10 mM Ca^2+^, with EhV replication and production dynamics being generally more muted in the latter (Fig. 4 and Supporting Information Fig. S3). Lower Ca^2+^ concentrations consistently yielded higher virus production in these strains (six‐fold for DHB611; 10‐fold for DHB607; Supporting Information Fig. S3). The timing of EhV production also differed between these host strains, with the majority of increase occurring at either 48 or 72 hpi for DHB607 or DHB611, respectively. Unlike DHB611, which displayed steady decreases in host cell abundance upon EhV addition, DHB607 cells grown in 10 mM Ca^2+^ did not experience substantial lysis. Rather, their cell abundance plateaued and only marginally declined after 48 hpi. While CCMP374 was confirmed to be naked at both Ca^2+^ concentrations (Figs 1 and 3 and Supporting Information Fig. S2), the dynamics of host cell lysis and EhV production dynamics were both enhanced under low Ca^2+^_._

DHB624 and DHB659 remained resistant to EhV86 infection at both 10 and 0.1 mM Ca^2+^ concentrations, indicating that they contain mechanisms of resistance independent of calcification. While the cellular mechanisms of resistance remain unknown in the *E. huxleyi*–EhV system, several naked strains display persistent resistance (Schroeder *et al*., 2002; Bidle *et al*., 2007; Bidle and Kwityn, 2012).

We examined the response of the most heavily calcified strain DHB607 at different calcification states to two different EhV strains, EhV86 and EhV207, the latter virus representing a more aggressive lytic strain with a shorter latency period (Nissimov *et al*., 2016). This allowed us to test whether the differential impact of calcification state on infectivity was specific to EhV strains or applies more broadly to *E. huxleyi*–EhV infection dynamics. Moderate resistance of calcified DHB607 cells grown at 10 mM Ca^2+^ through a 96 h infection period was observed when challenged with EhV86 (Fig. 4 and Supporting Information Fig. S5). Decreased growth rates compared to the uninfected control were observed with very little lytic burst and consistently low EhV production 96 hpi. Similar relative trends were observed when DHB607 cells were challenged with EhV207 (Supporting Information Fig. S4), with calcified cells grown at 10 mM Ca^2+^ displaying delayed infection dynamics, compared to the rapid lysis of DHB607 at 0.1 mM Ca^2+^. Unlike EhV86, notable lysis and reduction in host cell abundance were observed at 48 hpi, which was reflected in the enhanced production of EhV207 virus particles. Overall, EhV production was consistently lower in calcified DHB607 for both viruses (Supporting Information Figs S3 and S5).

### Linking sialic acid glycosphingolipids and calcification

**Figure 5:**
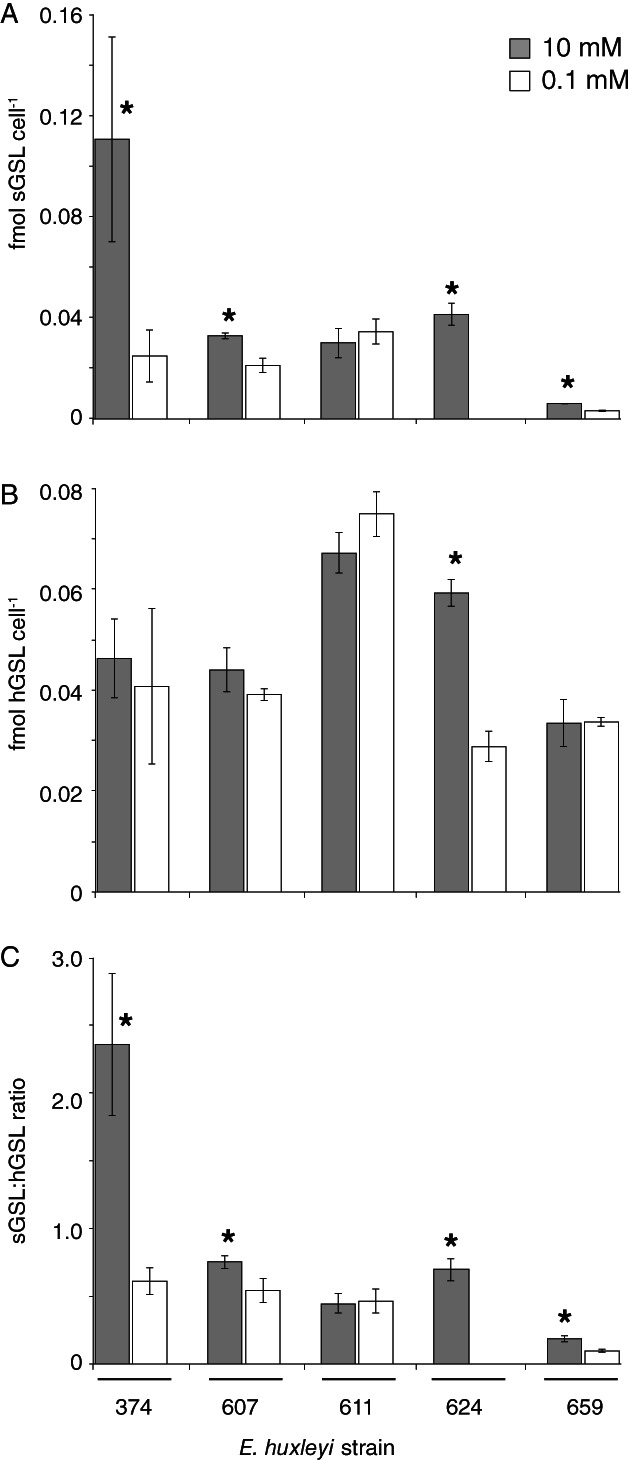
Glycosphingolipid composition of various *E. huxleyi* strains grown in defined ESAW media at different Ca^2+^ concentrations. The cell quotas of (A) sialic acid GSL (sGSL), (B) host GSL (hGSL) and (C) sGSL:hGSL ratio for host cells acclimated and grown under either 0.1 or 10 mM Ca^**2**+^ are shown. Both sGSLs and hGSLs represent distinct families of GSLs, with the former having a hypothesized connection to infectivity, Ca^**2+**^ and calcification (Fulton *et al*., 2014) and the later serving as a more general lipid biomarker for *E. huxleyi* cells (Vardi *et al*., 2012). Asterisks indicate statistical differences (*P* < 0.05; *n* = 3) between low and high Ca^2+^ conditions**.** Error bars denote standard deviation among biological triplicates. Where error bars are not visible, they are smaller than bar line thickness.

sGSLs are an important lipid biomarkers of EhV sensitivity (Fulton *et al*., 2014; Hunter et al., 2015) and are enriched in isolated lipid rafts from early‐infected(∼2 hpi) *E. huxleyi* cells (Rose *et al*., 2014). Fulton and colleagues (2014) proposed a biochemical relationship between sGSL and calcification, due to the specific binding properties of sialic acids for Ca^2+^ (Jaques *et al*., 1977). Our experimental approach of manipulating calcification state and sensitivity of distinct host strains allowed us to interrogate this proposed relationship. Alterations in Ca^2+^ concentration induced statistically significant (*P* < 0.05; *n* = 3) differences in the sGSL quota and sGSL:hGSL ratios, with 10 mM Ca^2+^, having generally elevated values (Fig. 5). Similar (within standard error of replicate measurements) cellular hGSL quotas were observed under both culture conditions, so Ca^2+^ concentration appeared to specifically impact the sGSL production. Intriguingly, naked strain CCMP374 had the most dramatic reduction in sGSL and sGSL:hGSL ratios when grown at 0.1 mM Ca^2+^, showing an additional impact of calcium availability on its production.

In order to establish a more direct link between sGSLs and calcification, we analysed the respective lipidomes of sorted coccoliths and sorted calcified cells from DHB607 and DHB624 (obtained via analytical flow cytometry and high‐speed cell sorting; see ‘Experimental procedures’ section; Supporting Information Fig. S6). These strains were the most heavily calcified and showed statistical differences in both PIC and sGSL quotas for cells grown at different Ca^2+^ concentrations (Figs 3 and 5). sGSLs were clearly detectable in sorted coccoliths of both host strains (Table 1) and were present at similar sGSL:hGSL ratios to that of sorted cells (Table 1). However, normalization of cell and coccolith sGSL quotas to their respective volumes (see ‘Experimental procedures’ section) showed a 3.9‐ and 3.4‐fold enrichment in the local sGSL concentration for coccoliths isolated from DHB607 and DHB624, respectively, compared to that for sorted cells, which could conceivably provide an enhanced adsorptive surface environment for EhVs.

**Table 1:** Lipid analysis of sorted cells and coccoliths.

Sample type	Strain	sGSL:hGSL	fmol sGSL μm^−3^ (normalized to cell volume)	fmol sGSL μm^−3^ (normalized to cocco lith volume)	Fold enrichment of sGSLs in coccoliths
Sorted cells	DHB607	1.17	5.53	**–**	**–**
Sorted coccoliths	DHB607	1.11	**–**	26.98	3.88
Sorted cells	DHB624	1.29	4.76	**–**	**–**
Sorted coccoliths	DHB624	1.52	**–**	20.85	3.38

The sGSL:hGSL ratios and sGSL concentrations are shown for DHB607 and DHB624, which include sorted cells and coccoliths. sGSL quotas have been normalized to cell and coccolith volume. Fold sGSL enrichment reflects the difference in concentration between cells and coccoliths.

To the best of our knowledge, this is the first time lipidomics that have been performed on calcified cells and detached coccoliths. These lipid species are likely acquired during formation within the coccolith vesicle (CV), an intracellular compartment derived from the Golgi body (Young *et al*., 1999). These vesicles, which have been well‐characterized in the coccolithophore species *Coccolithus pelagicus*, are closely associated to the nucleus, with the reticular and Golgi bodies flanking the opposite side (Taylor *et al*., 2007). Mature coccoliths dissociate from the nucleus and are exuded out onto the cell surface through the outer membrane in a single exocytotic event (Brownlee *et al*., 2015), a process that involves a fusion of the CV with the plasma membrane, before interlocking with other coccoliths forming the coccosphere (Taylor *et al*. 2007). It has been proposed that sGSLs could accompany coccoliths to the cell surface during the fusion event and may provide a scaffold for coccolith assembly (Westbroek *et al*., 1984; Fulton *et al*., 2014). While the proposed role of sGSL in the placement process is still speculative (Fulton *et al*. 2014), our data further link sGSLs and calcification. The specific roles of sGSLs on calcification and infectivity appear to be multifaceted.

### Mechanistic interplay between EhVs and calcification

**Figure 6:**
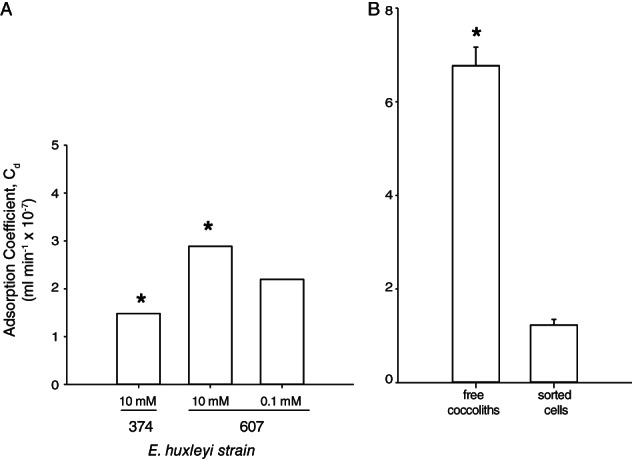
Adsorption of EhVs to *E. huxleyi* and detached coccoliths. (A) Measured adsorption coefficients for naked CCMP374, naked DHB607 and calcified DHB607 (*P* < 0.001, *n* = 3). (B) Measured adsorption coefficients for sorted coccoliths from calcified DHB607 compared to calcified DHB607 cells (*P* < 0.001, *n* = 3). Error bars represent calculated standard error (SD/$\sqrt{n}$) for triplicate measurements in one experiment. Panel (A) is shown with error bars, but the calculated standard error is smaller than bar line. Asterisks indicate statistical significance based on Student's *t*‐test.

Cellular calcification (and associated, detached coccoliths) would ostensibly provide a protective barrier to host–virus contact. Indeed, contact frequencies exert a first order control on host–virus interactions (Murray and Jackson, 1993; Thyrhaug *et al*., 2003; Danovaro *et al*., 2011; Short, 2012). At the same time, host–virus contact could be potentially facilitated in part by specific pools of GSLs (including sGSLs) and proteins that can bind to EhV‐specific moieties, like c‐type lectin domain‐containing proteins (Rose *et al*., 2014). Given that calcified phenotypes of DHB607, DHB624 and DHB659 had higher sGSL quotas (Fig. 5) and that purified coccoliths derived from DHB624 and DHB659 had elevated sGSLs concentrations (Table 1), we posited that this might enhance the successful adsorption of EhVs to host cells and facilitate infection. Of the three *E. huxleyi* host types tested, calcified DHB607 cells had the highest adsorption coefficient (*C*_d_) at 2.89 × 10^−7^ ml min^−1^ (Fig. 6A), followed by naked DHB607 (2.20 × 10^−7^ ml min^−1^). CCMP374 had the lowest *C*_d_ at 1.48 × 10^−7^ ml min^−1^ at an admittedly high sGSL quota. We caution that sGSL quota is not the only feature establishing adsorption coefficients. While we postulate that sGSLs play a role in facilitating the binding of EhVs, due to the presence of sGSLs in lipid rafts (Rose et al. 2014), several cellular features likely work in concert to determine adsorption coefficients, including strain dependency and other compositional properties of lipid rafts. Indeed, a unique class of GSLs, termed raft GSLs (rGSLs), were also enriched in purified lipid rafts (Rose *et al*., 2014) and are likely important for determining adsorption coefficients.

Perhaps most striking was the statistically higher (*P* < 0.001; *n* = 3) adsorption coefficients of purified, detached coccoliths from DHB607 (6.77 × 10^−7^ ml min^−1^) compared to calcified DHB607 cells (1.23 × 10^−7^ ml min^−1^; Fig. 6B). Normalization of *C*_d_ values to surface area showed the same comparative trend with free cocco liths having values of 6.51 × 10^−8^ ml min^−1^ μm^−2^ compared to 2.45 × 10^−9^ ml min^−1^ μm^−2^ for calcified DHB607. Our observations are consistent with coccoliths presenting a highly adsorptive reservoir for free viruses. Given coccoliths can outnumber cells by factors of ~ 17–26 in the environment (Balch *et al*., 1996), and viruses can outnumber hosts by a factor of 10 (Wommack and Colwell, 2000; Chibani‐Chennoufi *et al*., 2004), it suggests that coccoliths might effectively bind and sequester viable EhVs in natural populations owing both to enhanced contact frequencies and absorptive surface properties, thereby preventing contact with host cell membranes for infection. This is further supported by calcified strains having a higher frequency of collisions resulting in successful adsorption.

We calculated theoretical adsorption coefficients (*C*_td_, see ‘Experimental procedures’) using a value of 1 for frequency of collisions leading to adsorption (Brown and Bidle, 2014). Our theoretical adsorption coefficient (*C*_td_, 5.41 × 10^−11^ ml min^−1^) differed from our empirically measured values discussed above. By equating the theoretical value with our empirically determined adsorption coefficient and solving for the collision frequency, we found all treatments to have considerably higher collision numbers leading to successful adsorption. Calcified DHB607 had the highest number of collisions at 5340, which was 31% higher than naked DHB607 cells (4066) and ~ 95% higher than naked CCMP374 cells (2742). The elevated frequency of collisions afforded by detached coccoliths combined with their highly adsorptive properties presents a significant barrier to *E. huxleyi* host–virus interactions, rendering them inefficient.

### EhV‐induced shedding of coccoliths

**Figure 7:**
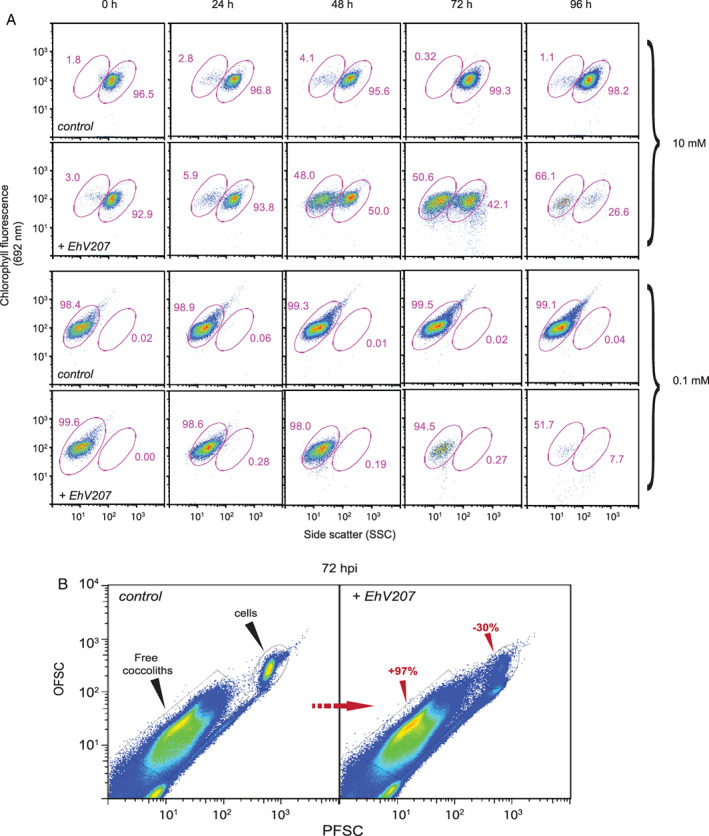
Dynamics of *E. huxleyi* calcification during EhV infection. (A) Time course of chlorophyll fluorescence (692 nm) and side scatter (SSC) for uninfected, control *E. huxleyi* cells (DHB607) and those challenged with EhV207. Cells were grown in ESAW with either 0.1 mM Ca^**2+**^ (naked cells) or 10 mM Ca^**2+**^ (calcified) and followed via flow cytometry for 96 hpi. Note the prominent shift to a lower SSC in the 10 mM Ca^2+^ cells at 24–48 hpi, indicative that a significant population of cells (~ 23%–45%) had shifted to a non‐calcified state. This is in contrast to cells grown in 0.1 mM Ca^2+^, which retain a very low SSC and are sensitive to infection (note decrease in cell number over the 96 h time period). Similar observations were seen when this same host was challenged with EhV86, with a somewhat delayed shift at 48–72 hpi (Supporting Information Fig. S8). (B) Relationship between PFSC light and OFSC light for EhV207‐infected cells at 72 hpi compared to controls. The former was characterized by prominent increases in detached coccoliths and reductions in calcified cells (indicated by red text and arrows).

In light of the enhanced adsorptive properties of detached coccoliths, we carefully monitored the dynamics of calcification and coccolith shedding during EhV infection using analytical flow cytometry and SSC. EhV207 infection of calcified DHB607 (10 mM Ca^2+^) induced a prominent shift in a subpopulation of resident cells to a lower SSC after 48 hpi (Fig. 7A); the geometric mean of SSC at 72 hpi was reduced by 55.6% (dropping from 95.2 down to 42.2) with the calcified populations represented 50.0% and 42.1% of host cells at 48 and 72 hpi respectively. In contrast, over ~ 95% of uninfected control cells retained a high SSC signature indicating they remained calcified. The lower SSC signature induced by EhV207 infection was consistent with that observed for naked *E. huxleyi* cells (Figs 1, 3 and 7) from CCMP374 and DHB607 (grown in 0.1 mM Ca^2+^), suggesting that this naked subpopulation was also sensitive to successful infection. This naked subpopulation eventually lysed ~ 96 hpi (Fig. 7A). Host cell growth halted after 48 hpi, but it was not accompanied by EhV production (data not shown).

The shift in SSC at 72 hpi of the host cells grown at 10 mM Ca^2+^ was accompanied by a 97% increase in detached coccoliths for the EhV207‐infected population compared to control at 72 hpi (Fig. 7B). SEM images visually confirmed the presence of detached coccoliths during EhV207 infection (data not shown). This EhV‐induced shift towards a higher percentage of naked cells at 72–96 hpi was consistent among replicates; statistically significant differences (*P* < 0.05, *n* = 3) were seen as early as 48 hpi between control and infected cultures (Supporting Information Fig. S7). Ultimately, the calcified DHB607 cells showed evidence of infection and lysis at 72–96 hpi as indicated by the smear of cell debris with lower chlorophyll content (692 nm). Importantly, similar SSC shifts in *E. huxleyi* calcification were also observed when DHB607 was challenged with EhV86, although they were delayed until 72–96 hpi (Supporting Information Fig. S8). This strongly suggested that the observed impact of EhV infection on calcification dynamics was not EhV strain specific.

Non‐calcifying DHB607 cells grown in 0.1 mM Ca^2+^ had a low SSC signature and consistently succumbed to infection, as is evidenced by the disappearance of *E. huxleyi* cells in the low SSC gate and the presence of a smear of low chlorophyll particles after 48 hpi (Fig. 7A) concomitant with host cell lysis (Supporting Information Fig. S4) and an increase in viral production after 48 hpi (Supporting Information Fig. S5). In both cases, the geometric mean of SSC remained in a reasonably tight cluster of around 11–17. Similar results were observed for EhV86‐challenged cells (Supporting Information Figs S5 and S8).

Our results show that enhanced calcification in DHB607 either prevents (in the case of EhV86) or delays (in the case of EhV207) successful infection, even though the presence of a coccosphere and detached coccoliths enhances the adsorption of EhVs. At the same time, the mere presence of EhVs themselves (absence of lysis) impacted cellular calcification dynamics by inducing ~ 85% lower SSC in a subpopulation of host cells (Fig. 7A and Supporting Information Fig. S7) to a level consistent with naked cells (SSC ~ 10–15) and the release of coccoliths into the water column during infection. The timing of this shift differed for EhV207 and EhV86, with the former inducing lower SSC in 48% and 56% of host cells at 48 and 72 hpi, respectively (Fig. 7A). Similar percentages of low SSC cell populations (27% and 58%) were induced by EhV86 but at 72 and 96 hpi, respectively (Supporting Information Fig. S8). We point out that this transition to naked cells was not an induced transition to 1 N haploid cells, as has been observed in previous work (Frada *et al*., 2008; 2012). The low SSC (naked) cells all have similar FSC (cell size) values which would be smaller for 1 N haploid cells. Furthermore, the coccolith shedding was observed for ~ 50% of the cell populations and at ~ 48–72 h post infection. For comparison, the observed percentage of 1 N haploid cells in the population was far lower (< 1%) and appeared a later time stage of infection, when most diploid cells had been lysed and removed.

Given the enhanced adsorption capacity of detached coccoliths (Fig. 6A), we posit that the massive shedding of coccoliths may be a cellular and mechanistic defence response whereby the adsorptive surface area is significantly increased, effectively sequestering viruses into an inaccessible reservoir. We confirmed that viable EhVs were necessary to induce the SSC shift response in calcified DHB607, as incubation of cells with either heat‐denatured or 0.02 μm pore size‐filtered virus lysates yielded neither infection (Supporting Information Fig. S9) nor a noticeable difference in SSC (data not shown).

### EhV‐induced elicitors manipulate calcification state

Given the SSC shift was observed upon infection of DHB607 prior to notable cell lysis, we hypothesized that a possible trigger of coccolith shedding might be host‐derived infochemicals produced upon interaction with EhVs. Cell‐signalling infochemicals have been shown to be effective at inducing cellular responses in marine phytoplankton, including induction of programmed cell death (PCD), upon sensing of a stress (Vardi *et al*., 2006; 2007; 2009). Isolated and purified vGSLs, the production of which critically regulate successful EhV infection of *E. huxleyi*, triggered PCD and mimicked successful infection when exogenously added to cell cultures at relevant concentrations to those measured during infection (Vardi *et al*., 2009). We reasoned that exposure of calcified *E. huxleyi* cells to 0.02 μm filtered ‘induced media’ (IM; derived from 72 hpi, EhV207‐infected, calcified DHB607 cultures in which a notable SSC shift had been detected via flow cytometry), would mimic the SSC shift and shedding of coccoliths and, in essence, demonstrate that EhV‐induced infochemicals can impact cellular PIC dynamics.

**Figure 8:**
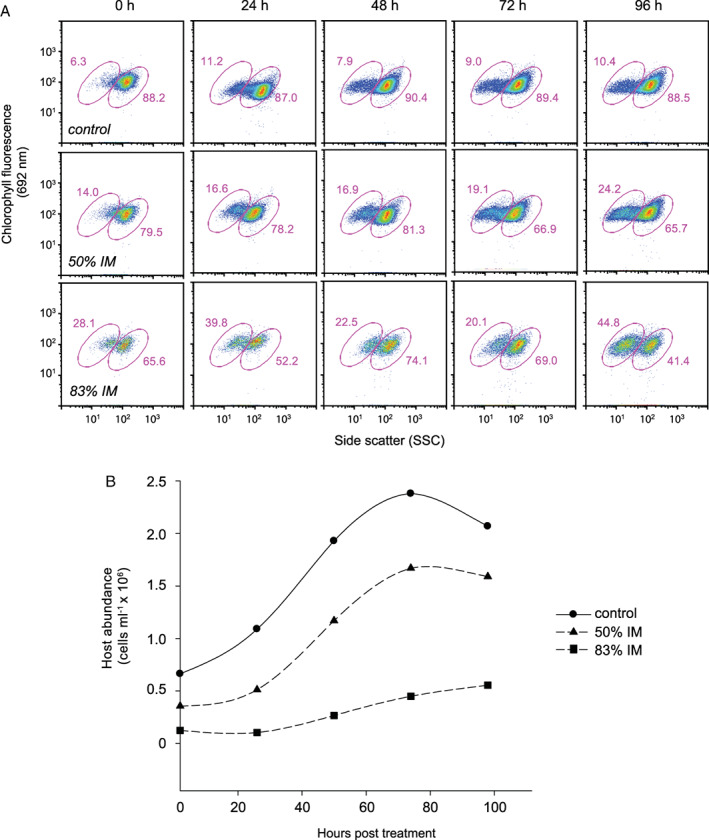
EhV‐induced media shifts host calcification. (A) Time course of chlorophyll fluorescence (692 nm) and side scatter (SSC) for uninfected *E. huxleyi* DHB607 cells that had been resuspended in different dilutions of ‘induced media’ (IM). IM was generated by infecting a calcifying culture (10 mM Ca^**2+**^) until a prominent shift in SSC was detected by flow cytometry (as per Fig. 7 at 72 hpi), at which point, all cells and viruses were removed by sequential filtration through 0.22 and 0.02 μm pore size filters. Healthy calcified cells were then resuspended in IM, and both chlorophyll and SSC dynamics were followed. Note that resuspension in 83% IM induced a similar shift in SSC by 72–96 h to that seen when cells were challenged with EhVs alone (Fig. 7 and Supporting Information Fig. S8), with no change in chlorophyll fluorescence. Control cells were resuspended in IM derived from uninfected control cells harvested at 72 h. (B) Time course of host abundance (measured by flow cytometry) for each aforementioned treatment. Note that no treatments resulted in the death of the cultures, but instead resulted in net growth, albeit to different levels and at different rates.

Exponentially growing DHB607 cells incubated in IM displayed a dose‐dependent response with both 50% and 83% IM stimulating higher numbers of cells with a ‘naked‐like’, low SSC signal at all time‐points (Fig. 8A). Comparisons between 83% IM‐treated samples and untreated controls at each respective time point revealed that 122%–346% more cells possessed low SSC, in response to dissolved elicitors in the IM. Both IM treatments induced a shift in 19%–20% of cells at 72 hpi, followed by shifts in 24% and 45%, respectively, at 96 hpi (Fig. 8A). These differential inductions of cells into a lower calcification state did not appear to be due to stress and death, as IM‐treated and control cultures displayed growth up through ~ 72 hpi into the incubation (Fig. 8B). Furthermore, the reductions in cell abundance in the control treatment at 96 h was not accompanied by a concomitant increase in coccolith shedding and the presence of naked cells (comprised 10.4% of the total); much higher coccolith shedding and proportion of naked cells was seen for the IM treatments. In fact, the 83% incubation displayed signs of continued growth, albeit slower, up through 96 hpi. This was also supported by the fact that all cell populations had robust and similar 692 nm chlorophyll signatures up through the 96 h incubation period and lacked low 692 nm debris indicative of dying cells. These observed changes in calcification state were likely not due to stress and death but rather point towards unidentified, host‐derived dissolved chemical elicitors. Given vGSLs were not detected in the dissolved (< 0.2 μm filtered) fraction (data not shown), they are likely not the coccolith shedding compounds. Rather, they are likely incorporated into the EhV virion, comprising ~ 63% of its lipidome (Vardi *et al*., 2009; Fulton *et al*., 2014). Purification and characterization of these chemical compounds are underway and may provide a new class of dissolved biomarkers for infection.

### Verifying virus–calcite interplay using naked and calcified phenotypes grown in the same media

We fortuitously generated a calcified phenotype of CCMP374 by long‐term culturing under P‐limiting conditions (see Experimental procedures). This strain has been in culture for ~ 50 years and was thought to have possibly lost this ability. The calcified phenotype was stable in f/2‐Si medium alongside the naked phenotype of CCMP374 allowing us to confirm our observations through side‐by‐side cellular measurements during infection experiments of the same strain in the same media. Calcified CCMP374 had a higher PIC quota [5.6 pg cell^−1^ *vs* ‘none detected’ in the naked phenotype (Supporting Information Fig. S10A)], CaCO_3_:POC ratio [0.35 *vs* < 0 (Supporting Information Fig. S10B)] and SSC geometric means (Supporting Information Fig. S11C). Calcified CCMP374 also had a slightly higher sGSL:hGSL ratio [5.2 compared to 4.9 for naked CCMP374 (Supporting Information Fig. S10C)], but they were not statistically significant. We note that these similar sGSL:hGSL ratios differ from the aforementioned stark differences in sGSL:hGSL ratios for CCMP374 grown in ESAW media with high and low calcium availability. These findings hint at a calcium‐associated feedback on sGSL regulation and production, which is consistent with their known biochemical affiliation. It is likely that the different levels of sGSL observed for cells grown in ESAW and f/2‐Si media reflect the differences in culturing conditions.

Calcified CCMP374 cells had significantly higher adsorption coefficients with EhV86 compared to their naked counterparts (*P* < 0.05, *n* = 3; Supporting Information Fig. S10D), with the respective *C*_d_ of 6.20 × 10^−8^ *versus* 3.81 × 10^−8^ ml^−1^ min^−1^, consistent with aforementioned findings and the idea that coccoliths can sequester EhVs and reduce successful encounters with the host. When comparing naked and calcified CCMP374, the calcified phenotype hade a ~ 63% higher collision frequency.

Calcification of CCMP374 also significantly impacted infection dynamics. Naked CCMP374 has been shown in the lab to be highly susceptible to infection with lysis occurring with 48–72 hpi (Schroeder *et al*., 2002; Bidle *et al*., 2007; Vardi *et al*., 2009; Bidle and Kwityn, 2012; Fulton *et al*., 2014; Kendrick *et al*., 2014). Calcified CCMP374 showed severely delayed infection dynamics with cells continuing to grow through 96 hpi (Supporting Information Fig. S11A), despite the production of EhVs (Supporting Information Fig. S11B). We also observed similar, massive shedding of detached coccoliths 72 hpi (Supporting Information Fig. S11C), as previously observed with calcified DHB607 (Fig. 7 and Supporting Information Fig. S8). These observations further support our conclusion that coccoliths provide temporal protection against EhV infection and that the presence of EhVs consistently induced the shedding of coccoliths across host strains.

## Conclusion

Our results demonstrate that calcification is another important factor influencing the dynamic arms race between *E. huxleyi* and coccolithoviruses. The fact that coccoliths serve as a primary ballast mechanism for associated POC in the oceans underscores the importance of this dynamic to carbon biogeochemistry. By altering the cellular calcification state without impacting cell growth physiology, we demonstrated that PIC content can determine EhV adsorption coefficients and replication. Calcification does indeed appear to play a protective role by serving as a physical barrier from, and adsorption reservoir for, virus particles. The enhanced EhV adsorption characteristics of detached coccoliths combined with their likely entrainment into TEP‐derived marine particles and aggregates (Laber *et al*., 2018; Nissimov *et al*., 2018; Sheyn *et al*., 2018) suggest that this might be an effective strategy to remove EhVs and function as an effective loss term. In this way, differential calcification states (coccolith morphologies and calcite content), arising from different genetic makeups, morphotype biogeographical patterns and nutrient availabilities, may critically structure epicentres of EhV infection in the ocean.

At the same time, the presence of EhVs themselves can lower the calcification state of host cells, effectively removing the protective barrier and facilitating infection. In this way, EhVs can induce an altered cellular state during active infection, whereby calcified cells are rendered naked through massive shedding of coccoliths simply in response to the presence of viable virus particles. Ultimately, this profound cellular and morphological change enhances host cell sensitivity to infection. Lastly, viable EhVs appear to trigger this calcification shift by inducing unidentified dissolved elicitors, consistent with the induction of infochemicals in resident populations. This suggests that EhVs can impact the carbon cycle in the absence of cell lysis. Given that EhVs are known to trigger TEP production and the formation of large, sinking aggregates during infection (Vardi *et al*., 2012; Nissimov *et al*., 2018), our findings have important implications to the carbon cycle by offering another caveat, whereby EhVs can trigger PIC flux. Our results contribute to the understanding of the impact of viruses in the marine environment and highlight their potential roles in inducing infochemical signalling and driving the biogeochemical cycling of carbon.

## Experimental procedures

### Culture growth and maintenance

*Emiliania huxleyi* strains DHB607, DHB611, DHB624 and DHB659 were obtained from the Plymouth Culture Collection of Marine Microalgae (http://www.mba.ac.uk/culture-collection/). These strains had been previously isolated via flow cytometry and single cell sorting by D. J. Hinz et al. (personal communication) during a 2008 mesocosm experiment conducted in Blomsterdalen, Norway (Vardi *et al*., 2012; Kimmance *et al*., 2014). CCMP374 and CCMP1516 were obtained from the National Center for Marine Algae and Micobiota (https://ncma.bigelow.org). All strains were grown in 250 ml plastic culture flasks (CELLSTAR Cell Culture Flasks with Filter Cap, Greiner Bio One) at 18°C under a 14:10 light:dark (*L*:*D*) cycle of 150 μmol photons m^−2^ s^−1^ in either ESAW artificial seawater medium or f/2‐Si seawater‐based medium (Guillard, 1975; Harrison *et al*., 1980; Berges *et al*., 2001). Naked CCMP374 was grown and acclimated to f/2‐Si at N:P 243:1 in continuous culture over a ~ 1 year period, during which time it maintained consistent calcification (as verified by flow cytometry SSC, PIC:POC measurements and SEM analysis). Calcified CCMP374 cells were subsequently transferred and maintained in replete f/2‐Si media and maintained calcification state for comparative analyses and infection experiments with naked CCMP374.

The EhV strains used in this study (EhV86 and EhV207) were previously isolated from *E. huxleyi* blooms in the English Chanel in 1999 and 2001, respectively (Wilson *et al*., 2002; Nissimov *et al*., 2012), and were obtained from the Plymouth Marine Laboratory virus collection in the UK. Virus strains were propagated in respective exponentially growing *E. huxleyi* CCMP374, DHB611 and DHB607 cultures, as previously described (Bidle *et al*., 2007). Lysates were filtered with 0.45 μm pore size syringe filters to remove debris and stored in the dark at 4°C until used for subsequent infection experiments.

### Scanning electron microscopy

Samples (100–500 μl) were vacuum filtered onto 0.22 μm pore size polyvinylidene fluoride (PVDF) filters (Durapore; Millipore, Sigma, Burlington, MA) and rinsed three to four times with an equal volume of MilliQ water (pH ~ 8) to remove any salts. Filters were dried for ~ 24 h and stored in a desiccator until imaged using a Phenom ProX bench‐top scanning electron microscope (ThermoFisherScientific, Eindhoven, The Netherlands). Image detection used the default, high‐sensitivity backscatter electron detector setting.

### Determination of PIC cellular quotas and CaCO_3_:POC ratios

A total of 5 ml of exponentially growing host cells (~ 5 × 10^5^ cells ml^−1^) were vacuum filtered (× 6) onto pre‐combusted GF/F filters using baked glassware (tower and stone filter base). The same volume of filtered seawater (0.45 μm pore size) was used to rinse the sides of the tower for each sample and ensure all biomass was collected. Additional filtered seawater was used to rinse the tower of any excess cells in between samples. Each GF/F filter was wrapped in pre‐combusted foil, labelled and stored in the dark at − 20°C until processing (~ 1 week). Filters were placed into numbered, etched glass petri dishes and baked at 60°C for drying. Triplicate filters from each culture were placed into a glass desiccator with concentrated HCl fumes for 24 h to dissolve PIC. The other set of triplicate filters was untreated and represented total carbon (TC). Triplicate sets [acidified (× 3) and non‐acidified (× 3)] were trimmed, packed into tin boats and combusted in a CNS Elemental Analyser (Carlo Erba NA 1500). PIC was determined by taking the difference between TC and POC acidified samples and dividing by the number of cells filtered. CaCO_3_:POC ratios were calculated dividing the cellular PIC quotas by cellular POC quotas.

### Determination of ESD values

Exponentially growing calcified and naked CCMP374 cells (~ 5 × 10^5^ cells ml^−1^) were diluted (1:50 in 0.45 μm‐filtered seawater and 0.5 ml were analysed using a Beckman Coulter Counter with a 70 μm orifice, using the mean particle size peak to determine ESD.

### Lipid analysis

Samples were filtered onto 25 mm diameter, 0.22 μm pore size Durapore PVDF membrane filters (GVWP type; Millipore), folded in pre‐combusted aluminium foil, snap frozen in liquid nitrogen and stored at − 80°C until processed. Lipids were extracted and characterized as previously described by (Fulton *et al*., 2014).

Quotas of sGSL for sorted cells and coccoliths were normalized to respective, post‐sort cell and coccolith concentrations and volume filtered for analysis. Respective cell and coccolith volumes were estimated to be 36.6 μm^3^ for cells and 0.3 μm^3^ for liths. Cell volume was estimated using *V* = 4/3*πr* ^3^, assuming a spherical shape for each cell, where *r* is a radius of ~ 2 μm. Coccolith volume was estimated using the standard density equation *D* = *m*/*v*, where *m* is the mass of a coccolith and *D* is the density of calcite. The mass of coccoliths (pg) was calculated using the equation *m* = 2.7 × *K*_s_ × *L* ^3^, where *K*_s_ is the shape dependent constant derived for normally calcified *E. huxleyi* cells (*K*_s_ = 0.02) and *L* is the coccolith length (~ 2.5 μm) (Young and Ziveri, 2000; Young *et al*., 2014). Mass was converted to volume using a density of 2.7 pg μm^−3^ for calcite (Young and Ziveri, 2000). These values are within published estimates of both cell and coccolith volume (Garde and Cailliau, 2000; Young and Ziveri, 2000; Young *et al*., 2014).

### Virus infection

All cultures were in exponential growth phase and grown to an abundance of ~ 5 × 10^5^ cells ml^−1^ prior to infection with *E. huxleyi* viruses [EhV86 or EhV207 (Wilson *et al*., 2005); (Nissimov *et al*., 2012)] at a virus:host ratio of 5. To verify the impact of viable EhVs on infection dynamics, cells were also inoculated with heat‐denatured (100°C, 10 min) and 0.02 μm pore‐size filtered (Anotop) lysates. Once infected, cultures were re‐sampled to determine initial viral abundance at *T*_0_. Subsamples were removed daily over the course of the experiment for host and virus analyses via flow cytometry.

### Flow cytometry analysis

Flow cytometry was conducted using an Influx Model 209S Mariner flow cytometer and high‐speed cell sorter equipped with a 488 nm 200 mW blue laser, 4‐way sort module, two scatter, two polarized and four fluorescence detectors (BD Biosciences, San Jose, CA). Analysis of cell abundance used chlorophyll fluorescence (692 nm**,** 40 nm band pass), forward scatter (FSC) and SSC with a pressure differential between the sample fluid and sheath fluid of 0.8–1 psi. SSC was analysed daily using a fixed gate and saved configuration to control for natural variation of the Influx. Laser alignment and size calibration were checked with 3 μm SPHERO Rainbow beads and the coefficient of variation (CV) in the 530 nm signal and FSC signals was always **<** 2%. Brewster angle optics were also used to measure depolarization of FSC light by cells and particles as previously described (Von Dassow *et al*., 2012). Brewster windows were oriented so that they either transmitted FSC light with polarization parallel to the sample stream [parallel polarized FSC (PFSC) light] and reflected FSC light polarized orthogonal to the sample stream [orthogonally polarized FSC (OFSC) light] or reflected FSC light with polarization parallel to the sample stream (PFSC), both going to individual photomultiplier detectors. Flow cytometry analyses were performed using the program FlowJo 8.8.7 (Ashland, OR).

Viral abundance was determined by staining with SYBR Gold (Life Technologies,ThermoFisher Scientific, Waltham, MA) and measurements of green fluorescence (520 nm**,** 40 nm band pass) as previously described (Brussaard, 2003). A total of 40 μl of sample were fixed with glutaraldehyde (0.5% final concentration) and stored for 15–30 min at 4°C, followed by flash freezing in liquid nitrogen and storage at − 80°C until further processing. Samples were then thawed, diluted 25‐fold in 0.22 μm‐filtered Tris/EDTA (TE) buffer (pH 8), stained with SYBR Gold (0.5–1× final concentration), incubated for 10 min at 80°C in the dark, cooled to RT for 5 min and mixed thoroughly prior to counting on the Influx. Viral abundance was analysed using a pressure differential (between sheath and sample fluid) of 0.7, resulting in a low flow rate for higher event rates.

### Adsorption assays

EhV adsorption to *E. huxleyi* host cells and detached coccoliths were determined empirically based on methods by Brown and Bidle (2014) and using triplicate, infected cultures at a virus:host ratio of 5 (data for EhV207 and EhV86 are presented in Fig. 6 and Supporting Information Fig. S10, respectively). Samples from each culture were fixed with glutaraldehyde (0.5% final concentration) at 0, 10, 30, 60, 120, 240 and 360 min post infection, and free virus counts were determined (as above). Adsorption coefficients (*C*_d_) were determined by plotting the natural logarithm of the fraction of free (i.e., unadsorbed) viruses against elapsed time. *C*_d_ (ml min^−1^) was calculated as *C*_d_ = *a*/*N*, where *a* is the slope of the regression line between the natural logarithm of the remaining fraction of free viruses plotted over time and N is the cell concentration (Cottrell and Suttle, 1995). Our analysis included a correction factor for virus adsorption to culture tubes in cell‐free controls (Bratbak *et al*., 1993; Murray and Jackson, 1993; Schroeder *et al*., 2002; Mann, 2003).

*C*_d_ values were also normalized to respective surface areas of detached coccoliths and cells using SA = 4*πr* ^2^ and using ~ 2 μm for the radius of cells. For coccoliths, we used the surface area equation for an ellipsoid, $\mathrm{SA}\;\approx \;4\pi \sqrt[{p}]{\frac{{\left(\mathrm{ab}\right)}^{p}+{\left(\mathrm{ac}\right)}^{p}+{\left(\mathrm{bc}\right)}^{p}}{3}}$, where *a* is half the coccolith length (~ 1 .25 μm), *b* is half the coccolith width (~ 1 μm) and *c* is the coccolith thickness (~ 0.5 μm). We used a value of 1.6 for (*p*), which is optimal for nearly spherical ellipsoids. This yielded values of 50.3 μm^2^ for cells and 10.4 μm^2^ for coccoliths, respectively, which were used to normalize calculated *C*_d_ values for free coccoliths and cells.

To determine theoretical adsorption coefficients, we first calculated the viral diffusion constant (*D*_v_) using *k*_B_ *T*/3*πμd*, where *k*_B_ is the Boltzmann constant, *T* is the temperature (18°C) in Kelvin, *μ* is the viscosity and *d* is the viral diameter (~ 180 nm). *D*_v_ (2.15 × 10^−8^ cm^2^ s^−1^) was used to calculate the theoretical adsorption coefficient (*C*_td_) using *C*_td_ = 4*πRdf*, where *R* is the cell radius (~ 2 μm), *d* is the viral diameter and *f* is the frequency of collisions resulting in adsorption. *C*_td_ can be calculated assuming an *f* value of 1 (Murray and Jackson, 1993; Mann, 2003; Brown and Bidle, 2014). Using our empirically measured *C*_d_, we can determine the frequency of collisions resulting in adsorption for each assay.

### IM incubations

Biological replicates (*n* = 2) of control and EhV86‐ or Eh207‐infected DHB607 cells (10 mM Ca^2+^) were incubated until 72 hpi, when a substantial shift in SSC was observed, at which point cultures were sequentially filtered through 0.22 μm pore size (Sterivex) to remove cells and any debris and 0.02 μm pore‐size (Anotop) filters to remove the virus particles. Calcified DHB607 cells (~ 5 × 10^5^ cells ml^−1^), which had been grown in ESAW containing 10 mM Ca^2+^, were then re‐suspended in 30 ml of IM at 50% and 83% strength; control cultures were re‐suspended in IM derived from uninfected control cells harvested at 72 h. Samples were analysed every 24 h via flow cytometry daily over a 96 h incubation period.

## Supporting information


**Fig. S1.** Growth of *E. huxleyi* strains.
Specific growth rates (*μ*; day^−1^) of *E. huxleyi* strains during exponential growth in ESAW with either 0.1 or 10 mM Ca^2+^ concentration (grey and white bars, respectively). Error bars represent standard error (SD/$\sqrt{n}$; *n* = 3) among biological replicates grown on different dates. Statistical significance was tested using Student's *t*‐test (*P* < 0.05, *n* = 3 for strains CCMP374, DHB607, DHB624 and DHB659). DHB611 was replicated twice; statistics are not provided but pattern is representative of both observations.

**Fig. S2.** Visualization of calcification.
Scanning electron microscopy (SEM) images of various strains of *E. huxleyi* cells grown in ESAW with either 0.1 or 10 mM Ca^2+^ concentration. Scale bars are indicated.

**Fig. S3.** EhV86 production dynamics for *E. huxleyi* cells grown at different calcification states.
EhV abundance from infection experiments of three different *E. huxleyi* host strains (CCMP374, DHB607 and DHB611) grown in 0.1 or 10 mM Ca^2+^ concentration (circles and triangles, respectively). Data correspond to host abundance dynamics presented in Fig. 4. Standard error for technical replicates (*n* = 3) was < 1%, which is smaller than symbol size.

**Fig. S4.** Comparative host–virus infection dynamics of *E. huxleyi* strain DHB607 at different calcification states when challenged with different EhVs.
Time course of host abundance for uninfected (closed symbols) and EhV infected (open symbols) *E. huxleyi* cells that were grown in ESAW under either 0.1 or 10 mM Ca^**2+**^. Note that two different EhV strains were used (EhV86 and EhV207) for the infection of two different hosts. High sensitivity to infection was displayed by those grown at low Ca^**2+**^, both of which were naked. Also, note the increased potency of EhV207, which can ultimately infect and begin to lyse calcified DHB607 after 96 hpi (also seen in Supporting Information Fig. S5). Error bars represent the standard deviation among triplicate measurements for one experiment, but were smaller than symbol size. Host virus dynamics shown were representative of experiments (*n* = 3) performed on different dates.

**Fig. S5.** EhV production dynamics for host cells grown at different calcification states.
Time course of EhV86 and EhV207 abundance for infected *E. huxleyi* DHB607 cells that were grown in ESAW with either 0.1 or 10 mM Ca^**2+**^.

**Fig. S6.** Visualization of sorted cells and free coccoliths.
SEM images of flow‐sorted cells (top image) and flow‐sorted detached coccoliths (bottom image). Scale bars are shown for reference.

**Fig. S7.** Dynamics of *E. huxleyi* calcification during infection with EhV207.
Time course of the percentage of calcified (A) and naked (B) *E. huxleyi* DHB607 cells, along with the abundance of detached coccoliths (C) for uninfected, control cells (open bars) and those challenged with EhV207 (closed bars). Cells were grown in ESAW containing 10 mM Ca^**2+**^ and were followed via flow cytometry for 96 hpi. Error bars represent the standard deviation from triplicate biological experiments. Asterisks indicate statistical significance based on Student's *t*‐test (*P* < 0.05, *n* = 3).

**Fig. S8.** Dynamics of *E. huxleyi* calcification during EhV infection.
Time course of chlorophyll fluorescence (692 nm) and side scatter (SSC) for uninfected, control *E. huxleyi* cells (DHB607) and those challenged with EhV86. Cells were grown in ESAW with either 0.1 mM Ca^**2+**^ (naked cells) or 10 mM Ca^**2+**^ (calcified) and followed via flow cytometry for 96 hpi. Note the prominent shift to a lower SSC in the 10 mM Ca^2+^ cells at 72–96 h, indicative that a significant population of cells (∼25%–50%) had shifted to a non‐calcified state. This is in contrast to cells grown in 0.1 mM Ca^2+^, which retain a very low SSC and are sensitive to infection (note decrease in cell number over the 96 h time period). Similar observations were seen when this same host was challenged with EhV207, with a somewhat earlier shift at 48–72 hpi (see Fig. 7).

**Fig. S9.** Host dynamics for EhV‐infected *E. huxleyi* at different calcification states.
Time course of host abundance for uninfected (blue triangles; solid line) and EhV207‐additions (other symbols, triangles; dotted lines) *E. huxleyi* cells that were grown under either 0.1 or 10 mM Ca^**2+**^. Infection of DHB607 was determined for intact, viable EhV207 virions (open triangles) compared to those that had been either heat‐denatured (solid green squares) or removed by 0.02 μm pore size filtration (solid orange diamonds). Standard error for technical replicates (*n* = 3) was < 1%, which is smaller than symbol size; host–virus dynamics shown were representative of experiments (*n* = 2) performed on different dates.

**Fig. S10.** Calcification state, glycosphingolipid composition, and adsorption coefficient for naked and calcified phenotypes of CCMP374.
(A) Cellular PIC quota, (B) CaCO_3_:POC and (C) sGSL:hGSL ratios for naked and calcified CCMP374 phenotypes grown in f/2‐Si. Error bars represent the standard error (SD/$\sqrt{n)}$ among triplicate measurements for one experiment. (D) Measured adsorption coefficients for EhV86 to naked and calcified CCMP374 phenotypes (*P* < 0.05, *n* = 3). Error bars represent calculated standard error for triplicate measurements in one experiment. The asterisk indicates statistical significance based on Student's *t*‐test.

**Fig. S11.** CCMP374‐EhV86 infection dynamics at altered calcification states.
(A) Time course of host abundance for uninfected (circles) and EhV86‐infected (triangles) naked (blue lines) and calcified (red lines) CCMP374 phenotypes grown in f/2‐Si. Error bars represent the standard error (SD/$\sqrt{n)}$ among triplicate measurements for one experiment.
(B) Time course of viral abundance for infected naked (blue lines) and calcified (red lines) shown in panel (A). Error bars represent the standard error among triplicate measurements for one experiment.
(C) Time course of chlorophyll fluorescence (692 nm) and side scatter (SSC) for EhV86‐infected naked and calcified CCMP374 phenotypes. Two time points were chosen to highlight the prominent shift from calcified to naked cells at 72 hpi, induced by the presence of EhV86 in calcified CCMP374.
