# Supplementary figures and images for: The mutual interplay between calcification and coccolithovirus infection
Source: Environ Microbiol. 2018 Sep 18;21(6):1896–915. doi: 10.1111/1462-2920.14362 (PMC7379532; doi:10.1111/1462-2920.14362)

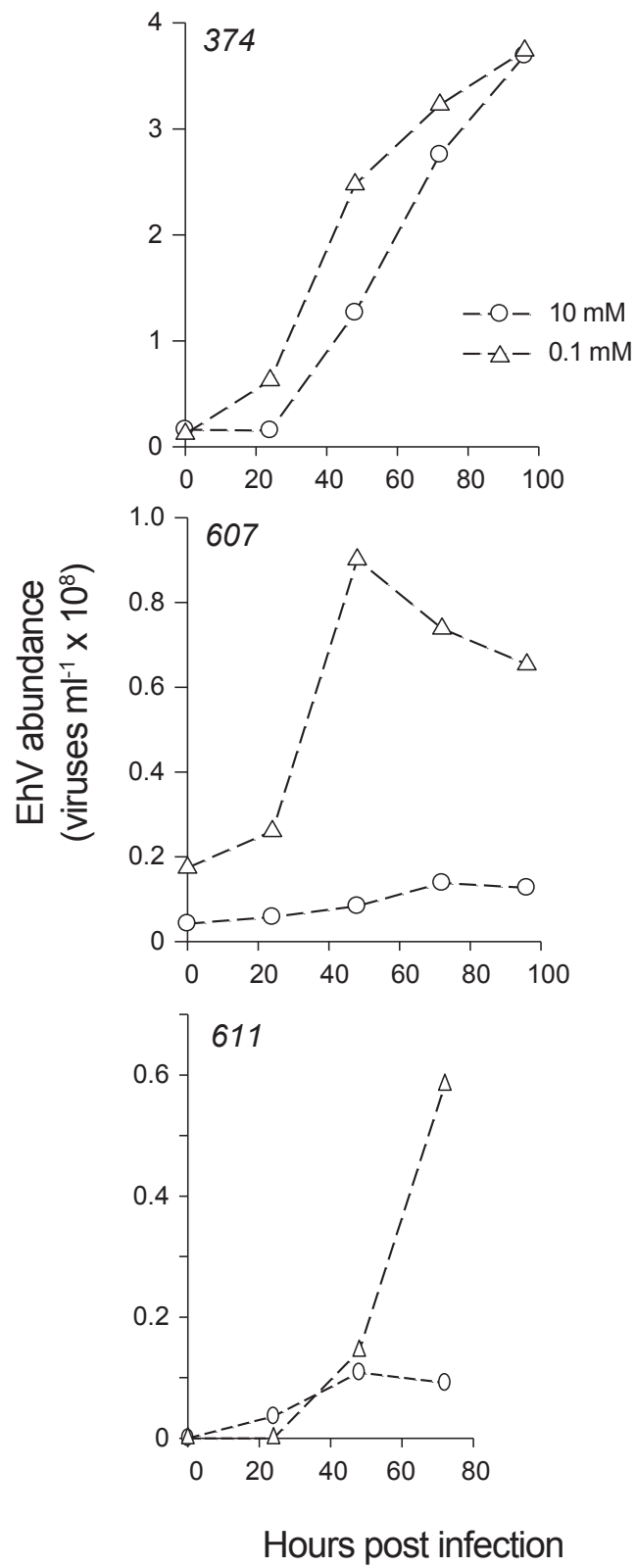

Figure S3, Johns et al.

Supplement: Supplementary file 3 — Fig. S3. EhV86 production dynamics for E. huxleyi cells grown at different calcification states. EhV abundance from infection experiments of three different E. huxleyi host strains (CCMP374, DHB607 and DHB611) grown in 0.1 or 10 mM Ca2+ concentration (circles and triangles, respectively). Data correspond to host abundance dynamics presented in Fig. 4. Standard error for technical replicates (n = 3) was < 1%, which is smaller than symbol size. [file EMI-21-1896-s003.pdf]
